# Community health workers trained to conduct verbal autopsies provide better mortality measures than existing surveillance: Results from a cross-sectional study in rural western Uganda

**DOI:** 10.1371/journal.pone.0211482

**Published:** 2019-02-13

**Authors:** Doreen Nabukalu, Moses Ntaro, Mathias Seviiri, Raquel Reyes, Matthew Wiens, Radhika Sundararajan, Edgar Mulogo, Ross M. Boyce

**Affiliations:** 1 Department of Community Health, Mbarara University of Science & Technology, Mbarara, Uganda; 2 Department of Health, Wakiso District Local Government, Kampala, Uganda; 3 Division of General Medicine & Clinical Epidemiology, University of North Carolina at Chapel Hill, Chapel Hill, United States of America; 4 Center for International Child Health, BC Children’s Hospital, Vancouver, BC, Canada; 5 Department of Emergency Medicine, Weill Cornell Medical Center, New York, NY, United States of America; 6 Division of Infectious Diseases, University of North Carolina at Chapel Hill, Chapel Hill, United States of America; University of the Western Cape, SOUTH AFRICA

## Abstract

**Background:**

In much of sub-Saharan Africa, health facilities serve as the primary source of routine vital statistics. These passive surveillance systems, however, are plagued by infrequent and unreliable reporting and do not capture events that occur outside of the formal health sector. Verbal autopsies (VA) have been utilized to estimate the burden and causes of mortality where civil registration and vital statistics systems are weak, but VAs have not been widely employed in national surveillance systems. In response, we trained lay community health workers (CHW) in a rural sub-county of western Uganda to conduct VA interviews in order to assess the feasibility of leveraging CHW to measure the burden of disease in resource limited settings.

**Methods and findings:**

Trained CHWs conducted a cross-sectional survey of the 36 villages comprising the Bugoye sub-county to identify all deaths occurring in the prior year. The sub county has an estimated population of 50,249, approximately one-quarter of whom are children under 5 years of age (25.3%). When an eligible death was reported, CHWs administered a WHO 2014 VA questionnaire, the results of which were analyzed using the InterVA-4 tool. To compare the findings of the CHW survey to existing surveillance systems, study staff reviewed inpatient registers from neighboring referral health facilities in an attempt to match recorded deaths to those identified by the survey. Overall, CHWs conducted high quality VA interviews on direct observation, identifying 230 deaths that occurred within the sub-county, including 77 (33.5%) among children under five years of age. More than half of the deaths (123 of 230, 53.5%) were reported to have occurred outside a health facility and thus would not be captured by passive surveillance. More than two-thirds (73 of 107, 68.2%) of facility deaths took place in one of three nearby hospitals, yet only 35 (47.9%) were identified on our review of inpatient registers. Consistent with previous VA studies, the leading causes of death among children under five years of age were malaria (19.5%), prematurity (19.5%), and neonatal pneumonia (15.6%). while among adults, HIV/AIDS-related deaths illness (13.6%), pulmonary tuberculosis (11.4%) and malaria (8.6%) were the leading causes of death. No child deaths identified from inpatient registers listed HIV/AIDS as a cause of death despite 8 deaths (10.4%) attributed to HIV/AIDS as determined by VA.

**Conclusions:**

Lay CHWs are able to conduct high quality VA interviews to capture critical information that can be analyzed using standard methodologies to provide a more complete estimate of the burden and causes of mortality. Similar approaches can be scaled to improve the measurement of vital statistics in order to facilitate appropriate public health interventions in rural areas of sub-Saharan Africa.

## Introduction

Despite longstanding efforts to improve epidemiological surveillance, most sub-Saharan African countries still face challenges associated with identifying communicable disease outbreaks and reporting routine vital statistics [1, 2]. The lack of accurate and up-to-date information regarding the number, location, and causes of death negatively impacts the nature and timing of subsequent policy responses [3]. Improvements in existing surveillance systems, which are primarily facility-based, are unlikely to yield comprehensive data, as many births and deaths in rural sub-Saharan Africa occur outside of health facilities, especially among the most vulnerable [4, 5]. These information gaps thus contribute to underreporting of infectious diseases of poverty and other health conditions that disproportionately impact the poorest and most isolated segments of society. The Ebola virus outbreak that occurred in West Africa between 2014 and 2016 highlighted these vulnerabilities and demonstrated how an epidemic can proliferate with enormous socio-economic disruption in the absence of strong health systems capable of a rapid and integrated response [6, 7].

In Uganda, like many countries in sub-Saharan Africa, health facilities serve as the primary data collection sites for routine health statistics. Each month, individual patient information is aggregated and forwarded to the respective District Health Office and to the Ministry of Health [8]. This passive surveillance system is plagued by a number of issues, including inadequate capacity, weak and irregular monitoring, and reporting bias, all of which negatively affect the validity and thus utility of the data [9, 10]. Although civil registration with high and representative coverage should be the long-term goal, investment in complementary, interim sources of statistics are urgently needed to accelerate the design, implementation, and evaluation of cost-effective interventions and meet the Sustainable Development Goal of 100% birth and 80% death registration [5, 11, 12].

Verbal autopsies (VA) have been adopted as a practical means of determining the cause of death based on an interview with the next of kin or caregiver of the deceased in areas where civil registration and vital statistics (CRVS) systems are weak [13–15]. Multiple studies have demonstrated that non-physician, mid-level providers can conduct the verbal autopsies with a reasonable estimate of the cause of death [16–18]. Some studies have also shown that community health workers (CHW) can successfully carry out VA interviews using simplified questionnaires [19, 20]. In Uganda, community health workers, known as village health teams (VHTs), are lay volunteers who are members of the communities where they work, are selected by the communities and are answerable to the communities for their activities. They form the first health unit according to Uganda’s health sector structure with a wide range of functions including disease surveillance, record keeping, collecting of data on vital events and data management among others [21]. However, standardized methods and reporting structures are not well defined. Surveillance and mortality data from village health teams continues to be scanty or unavailable [22]. Because of their trusted status in the community, we hypothesized that VHTs would be uniquely positioned to identify deaths and conduct VA interviews. Therefore, the objective of this study was to assess the feasibility of leveraging CHW to measure the burden of disease in resource limited settings.

## Methods

### Study setting

The study was conducted in Bugoye sub-county in the Kasese District of western Uganda (0^o^ 18’ North, 30^o^ 5’ East). The sub-county is comprised of five parishes and 36 villages spanning an area of 55 km^2^ with an estimated population of 50,249, approximately one-quarter of whom are children under 5 years of age (25.3%) [23]. There are nearly 7,000 households in the sub-county, with an average size of 7.3 persons per household.

The terrain in the sub-county is rugged and highly varied. Village elevations range from 1,100m along the river basins to upwards of 1,800m near the western border with the Rwenzori National Park. The area is predominantly rural and most residents work as subsistence farmers. Road access in many villages is limited, especially during the rainy seasons. The main means of transport is via motorcycle taxi, although some villages can only be reached on foot.

Although no sub-county specific data is available, it is assumed that like much of Uganda, malaria, along with respiratory illnesses and diarrheal diseases, accounts for the majority of deaths in children under five years of age. Local adult mortality statistics, including those describing maternal mortality, are also limited to generalizations drawn from national surveys.

There are seven public health facilities distributed throughout Bugoye sub-county. These include five Level II Health Centers staffed by nurses and midwives, and two Level III Health Centers, which are led by a clinical officer, and include inpatient and labor and delivery wards, in addition to limited laboratory services. The nearest referral hospital is Kilembe Mines Hospital, which is a private not-for-profit facility located approximately 25 km from the sub-county. Public transport to Kilembe Mines Hospital typically takes 45 minutes at costs that can represent the weekly income for a typical subsistence farmer.

### Study design

This was a population-based, cross-sectional study of Bugoye sub-county to assess the feasibility of leveraging CHW to measure the burden of disease in resource-limited settings using a validated WHO 2014 verbal autopsy tool.

Trained VHTs surveyed all households in their respective areas of responsibility to identify those households where a death occurred in the one-year period between January 1, 2016 and December 31, 2016. Verbal autopsy results were analyzed with the InterVA model to estimate the cause of death [24, 25]. Results of the survey were compared to routinely collected records from the Kasese District Health Office and clinical records from the nearest referral centers.

### Selection and training of VHTs

One VHT member with at least one year of secondary education, and competent in both reading and writing, was identified by the VHT Coordinator at BHCIII from each of the 36 villages of Bugoye sub county. The thirty-six VHTs were trained for one week at Bugoye Health Center III (BHC) in the conduct of verbal autopsy interviews, conducted in February 2018 and led by the first author. During the training, Trainers reviewed all elements of the questionnaire, as well as the flow, meaning, and purpose of each question with the participants The VHTs also learned about the cardinal signs and symptoms, Description of the different diseases in the local language, community entry skills among others, the Techniques utilized in the training sessions included lectures, group discussions, practical exercises, and role play. The trainers conducted quality assurance exercises including direct observation of VA interviews and inspection of completed questionnaires to confirm adherence to study protocols and ensure the completeness and accuracy of the data. Approximately 20% of the VHT interviews were directly observed by the trainers.

### Identification of deaths in the village

In rural settings of Uganda, the village council chairman (LC) and respective VHT are generally invited to attend every burial ceremony in their village. Using this information, the VHTs in collaboration with the LCs, identified all households where a death occurred in the preceding twelve months. A next of kin or caretaker who was present with the deceased was identified and an appointment made for the interview. If a household had multiple deaths, data was collected on each individual death in that household using a separate survey.

### Conducting of verbal autopsy interviews

For the households which had reported a death and an appointment had been made, a trained VHT of that respective village conducted a verbal autopsy interview using a WHO 2014 VA questionnaire with the primary caregiver.

### Data collection tool /questionnaire

WHO 2014 VA questionnaires [24] were used to collect data on the identified deaths. The questionnaire captures information relevant to assessment of causes of death and the context including demographic information of the deceased, medical history, general signs, symptoms, risk factors, service utilization and reported cause of death. This tool also includes an open narrative text field.

### Passive surveillance

In an attempt to capture information on deaths that occurred in referral health facilities outside of the sub-county, we also reviewed the records of three health facilities where the majority of the facility deaths were reported to have occurred. These included Kilembe Mines Hospital, Kagando Hospital, and St. Paul’s Level IV Health Center. Using basic demographic data (i.e. age, sex, and village of residence) we attempted to identify any deaths from Bugoye sub-county and link them to those identified in the VHT survey.

### Statistical analysis

Data from VA interviews was assessed for quality and extracted from the questionnaire into an EpiData database (EpiData Association, Odense, Denmark). The results were analyzed using the InterVA4 (www.interva.net) to estimate the cause of death from verbal autopsy [25]. The InterVA-4 computer program was chosen because it presents a relatively simple, fast, cheap, very accessible, do not require training of physicians to review and ideal for determining cause of death on a large scale. Cause. Specific mortality fractions were determined as the proportion of all deaths that were attributable to any specific cause of death. We stratified mortality analysis by age. All statistical analysis was carried out using Stata 14 (College Station, TX).

### Ethical considerations

Ethical approval of the study was provided by the institutional review committee of the Mbarara University of Science and Technology and the Uganda National Council for Science and Technology. A Meeting was held with the DHO, sub county leaders and LC1 chairmen and verbal ascent was obtained on behalf of the community after the researcher gave them a presentation about the study, In the villages the VHTs consented the participants by briefing them on the objectives of the study and procedure, benefits for participating, what happens in case one does not participate, contacts in case of a problem arising from the study among others. After the brief, participants who verbally consented were requested to sign or put a thumb print on the consent form. All the participants consented.

## Results

### Household survey & verbal autopsies

The VHT-led household survey identified a total of 243 deaths occurring during the pre-specified study period. Of the identified deaths, 13 (5.3%) were stillbirths and excluded from the analysis. The remaining 230 deaths were included in the analysis.

The age and sex distribution of the 230 deaths included in our analysis is shown in **Table 1**.

**Table 1:** Age and sex distribution of deaths identified by VHT-led household survey.

	All deaths, n = 230		Sex			
			Male, n = 137 (59.6%)		Female, n = 93 (40.4%)	
**Age group**	n	%	n	%	n	%
Neonate	38	16.52	26	18.98	12	12.90
1–11 months	21	9.13	12	8.76	9	9.68
1–4 years	18	7.83	8	5.84	10	10.75
5-14years	13	5.65	8	5.84	5	5.38
15–49 years	65	28.26	36	26.28	29	31.18
50–65 years	23	10.00	17	12.41	6	6.45
>65 years	52	22.61	30	21.9	22	23.66

More than a quarter (n = 59, 25.7%) of reported deaths took place in the first year of life, and more than one-third (n = 77, 33.5%) occurred before the age of five years. Overall, there were more deaths among males (n = 137, 59.6%) even though they comprise less than half of the sub-county population (47.9%). The vast majority of respondents (211 of 230, 91.7%) reported living more than thirty minutes from the nearest health facility, and more than half of deaths (123 of 230, 53.5%) occurred outside a health facility.

For children under five years of age, the leading causes of mortality were malaria (19.5%), prematurity (19.5%), and neonatal pneumonia (15.6%), while among adults, HIV/AIDS-related illness (13.6%), pulmonary tuberculosis (11.4%) and malaria (8.6%) were the leading causes of death. Notably, when combined, various cancers (i.e. digestive, respiratory, reproductive) accounted for a substantial number of deaths among adults (24 of 230, 10.4%). (**Table 2**)

**Table 2:** Causes specific mortality fractions by age.

	All age		Under 5		Age 5–14		Adults (15 + yrs)	
	N = 230		N = 77 (33.48%)		N = 13 (5.65%)		N = 140 (60.87%)	
Cause of death (WHO 2012 VA model)	n	%	n	%	n	%	n	%
01.03 HIV/AIDS related death	30	13.0	8	10.4	3	23.1	19	13.6
01.05 Malaria	29	12.6	15	19.5	2	15.4	12	8.6
01.09 Pulmonary tuberculosis	16	7.0	0	0.0	0	0	16	11.4
10.01 Prematurity	15	6.5	15	19.5	0	0	0	0.0
12.09 Assault	14	6.1	0	0.0	0	0	14	10.0
01.02 Acute resp infect incl pneumonia	13	5.6	7	9.1	0	0	6	4.3
04.02 Stroke	13	5.6	0	0.0	0	0	13	9.3
10.03 Neonatal pneumonia	12	5.2	12	15.6	0	0	0	0.0
02.02 Digestive neoplasms	10	4.4	0	0.0	1	7.7	9	6.4
04.99 Other and unspecified cardiac dis	7	3.0	0	0.0	0	0	7	5.0
06.01 Acute abdomen	7	3.0	2	2.6	2	15.4	3	2.1
02.05 & 02.06 Reproductive neoplasms	6	2.6	0	0.0	0	0	6	4.3
02.03 Respiratory neoplasms	4	1.7	0	0.0	0	0	4	2.9
02.99 Other and unspecified neoplasms	4	1.7	0	0.0	0	0	4	2.9
12.08 Intentional self-harm	4	1.7	0	0.0	0	0	4	2.9
01.04 Diarrhoeal diseases	3	1.3	3	3.9	0	0	0	0.0
01.07 Meningitis and encephalitis	5	2.2	2	2.6	2	15.4	1	0.7
01.99 Other and unspecified infect dis	3	1.3	0	0.0	0	0	3	2.1
03.02 Severe malnutrition	4	1.7	2	2.6	1	7.7	1	0.7
04.01 Acute cardiac disease	3	1.3	0	0.0	0	0	3	2.1
04.03 Sickle cell with crisis	3	1.3	2	2.6	0	0	1	0.7
10.02 Birth asphyxia	3	1.3	3	3.9	0	0	0	0.0
03.03 Diabetes mellitus	2	0.9	0	0.0	0	0	2	1.4
06.02 Liver cirrhosis	2	0.9	0	0.0	0	0	2	1.4
08.01 Epilepsy	2	0.9	0	0.0	0	0	2	1.4
09.04 Obstetric haemorrhage	2	0.9	0	0.0	0	0	2	1.4
10.06 Congenital malformation	2	0.9	2	2.6	0	0	0	0.0
10.99 Other and unspecified neonatal death	2	0.9	2	2.6	0	0	0	0.0
12.01 Road traffic accident	2	0.9	0	0.0	0	0	2	1.4
03.01 Severe anaemia	1	0.4	0	0.0	1	7.7	0	0.0
05.01 Chronic obstructive pulmonary dis	1	0.4	0	0.0	0	0	1	0.7
07.01 Renal failure	1	0.4	0	0.0	0	0	1	0.7
10.04 Neonatal sepsis	1	0.4	1	1.3	0	0	0	0.0
12.04 Accid drowning and submersion	1	0.4	1	1.3	0	0	0	0.0
12.05 Accid expos to smoke fire & flame	2	0.9	0	0.0	1	7.7	1	0.7
99 Indeterminate	1	0.4	0	0.0	0	0	1	0.7

There were differences in the distribution of causes of deaths which were reported to have occurred at home and those reported to have occurred at health facility. For example, among children under five, HIV/AIDS related causes were more common among deaths that occurred at home compared to those that occurred in a health facility (18.2% vs. 4.6%, p = 0.07) (**Table 3).** Among adults, deaths attributed to assault occurred more frequently outside of health facilities (15.7% vs. 1.8%, p = 0.004) (**Table 4**). Deaths due to malaria, one of the leading causes of morbidity, occurred with similar frequencies between home and health facilities both among children (21.2% vs. 18.2%, p = 0.78) and adults (9.6% vs. 7.0%, p = 0.56).

**Table 3:** Comparison of the causes of deaths among children under five years who died in health facilities and those who died at home as reported by the verbal autopsy.

	Deaths Under five.					
	Total, N = 77		Death at facility n = 44		Death outside facility n = 33	
**InterVA-4 cause (WHO 2012 categories)**	n	%	n	%	n	%
01.02 Acute resp infect incl pneumonia	7	9.1	5	11.4	2	6.1
01.03 HIV/AIDS related death	8	10.4	2	4.6	6	18.2
01.04 Diarrhoeal diseases	3	3.9	1	2.3	2	6.1
01.05 Malaria	15	19.5	8	18.2	7	21.2
01.07 Meningitis and encephalitis	2	2.6	2	4.6	0	0.0
03.02 Severe malnutrition	2	2.6	1	2.3	1	3.0
04.03 Sickle cell with crisis	2	2.6	1	2.3	1	3.0
06.01 Acute abdomen	2	2.6	1	2.3	1	3.0
10.01 Prematurity	15	19.5	8	18.2	7	21.2
10.02 Birth asphyxia	3	3.9	3	6.8	0	0.0
10.03 Neonatal pneumonia	12	15.6	8	18.2	4	12.1
10.04 Neonatal sepsis	1	1.3	1	2.3	0	0.0
10.06 Congenital malformation	2	2.6	1	2.3	1	3.0
10.99 Other and unspecified neonatal	2	2.6	2	4.6	0	0.0
12.04 Acid drowning and submersion	1	1.3	0	0.0	1	3.0

**Table 4:** Comparison of the causes of deaths among adults for the deaths reported to have occurred in health facilities and those that occurred at home.

InterVA-4 cause (WHO 2012 categories)	Adult deaths.					
	≥15, n = 140		Death at facility N = 57		Death outside facility N = 83	
	n	%	n	%	n	%
01.02 Acute resp infect incl pneumonia	6	4.3	3	5.3	3	3.6
01.03 HIV/AIDS related death	19	13.6	9	15.8	10	12.0
01.05 Malaria	12	8.6	4	7.0	8	9.6
01.07 Meningitis and encephalitis	1	0.7	1	1.8	0	0.0
01.09 Pulmonary tuberculosis	16	11.4	7	12.3	9	10.8
01.99 Other and unspecified infect dis	3	2.1	1	1.8	2	2.4
02.02 Digestive neoplasms	9	6.4	4	7.0	5	6.0
02.03 Respiratory neoplasms	4	2.9	1	1.8	3	3.6
02.05 & 02.06 Reproductive neoplasms	6	4.3	3	5.3	3	3.6
02.99 Other and unspecified neoplasms	4	2.9	2	3.5	2	2.4
03.02 Severe malnutrition	1	0.7	0	0.0	1	1.2
03.03 Diabetes mellitus	2	1.4	1	1.8	1	1.2
04.01 Acute cardiac disease	3	2.1	2	3.5	1	1.2
04.02 Stroke	13	9.3	4	7.0	9	10.8
04.03 Sickle cell with crisis	1	0.7	1	1.8	0	0.0
04.99 Other and unspecified cardiac dis	7	5.0	5	8.8	2	2.4
05.01 Chronic obstructive pulmonary dis	1	0.7	1	1.8	0	0
06.01 Acute abdomen	3	2.1	1	1.8	2	2.4
06.02 Liver cirrhosis	2	1.4	0	0.0	2	2.4
07.01 Renal failure	1	0.7	0	0.0	1	1.2
08.01 Epilepsy	2	1.4	1	1.8	1	1.2
09.04 Obstetric haemorrhage	2	1.4	2	3.5	0	0
12.01 Road traffic accident	2	1.4	1	1.8	1	1.2
12.05 Accid expos to smoke fire & flame	1	0.7	0	0.0	1	1.2
12.08 Intentional self-harm	4	2.9	2	3.5	2	2.4
12.09 Assault	14	10.0	1	1.8	13	15.7
99 Indeterminate	1	0.7	0	0.0	1	1.2

### Passive surveillance

Of the 107 respondents who reported the death of a household member in a health facility, 73 (68.2%) were reported to have occurred in one of the three referral facilities in the district. A subsequent review of inpatient registers at these facilities identified 35 deaths of Bugoye sub-county residents, representing 47.9% of the total number of deaths which were reported to have occurred in these facilities as per the VHT household survey. More than half (19 of 35, 54.3%) of the deaths were recorded at Kilembe Mines Hospital, with the remainder from Kagando Hospital (9 of 35, 25.7%) and St. Paul’s Level IV Health Center (7 of 35, 20%).

According to the inpatient registers, the leading causes of death among hospitalized children under five years of age (n = 15), were malaria, prematurity, and neonatal pneumonia. This trend is similar to those determined by the verbal autopsies. Notably, no child deaths identified from inpatient registers listed HIV/AIDS as a cause of death, despite 8 deaths (10.4%) attributed to HIV/AIDS as determined by verbal autopsy. Among adults (n = 20), there was much less agreement between the inpatient registers and VA findings (**Table 5**).

**Table 5:** Comparison of the reported causes of deaths among children under five years and adults as determined by verbal autopsy and healthy-facility records.

	Under 5				Adults (15 + yrs)			
	Facility records		Verbal autopsy		Facility records		Verbal autopsy	
	N = 15		N = 77		N = 20		N = 140	
Cause of death (WHO 2012 VA model)	n	%	n	%	n	%	n	%
01.03 HIV/AIDS related death	0	0.0	8	10.4	3	15	19	13.6
01.05 Malaria	4	26.7	15	19.5	1	5	12	8.6
01.09 Pulmonary tuberculosis	0	0.0	0	0.0	2	10	16	11.4
10.01 Prematurity	4	26.7	15	19.5	0	0.0	0	0.0
12.09 Assault	0	0.0	0	0.0	0	0.0	14	10.0
01.02 Acute resp infect incl pneumonia	0	0.0	7	9.1	3	15	6	4.3
04.02 Stroke	0	0.0	0	0.0	0	0.0	13	9.3
10.03 Neonatal pneumonia	3	20.0	12	15.6	0	0.0	0	0.0
02.02 Digestive neoplasms	0	0.0	0	0.0	0	0.0	9	6.4
04.99 Other and unspecified cardiac dis	0	0.0	0	0.0	0	0.0	7	5.0
06.01 Acute abdomen	1	6.7	2	2.6	0	0.0	3	2.1
02.05 & 02.06 Reproductive neoplasms	0	0.0	0	0.0	0	0.0	6	4.3
02.03 Respiratory neoplasms	0	0.0	0	0.0	0	0.0	4	2.9
02.99 Other and unspecified neoplasms	0	0.0	0	0.0	0	0.0	4	2.9
12.08 Intentional self-harm	0	0.0	0	0.0	0	0.0	4	2.9
01.04 Diarrhoeal diseases	0	0.0	3	3.9	0	0.0	0	0.0
01.07 Meningitis and encephalitis	0	0.0	2	2.6	0	0.0	1	0.7
01.99 Other and unspecified infect dis	0	0.0	0	0.0	0	0.0	3	2.1
03.02 Severe malnutrition	0	0.0	2	2.6	0	0.0	1	0.7
04.01 Acute cardiac disease	0	0.0	0	0.0	0	0.0	3	2.1
04.03 Sickle cell with crisis	0	0.0	2	2.6	0	0.0	1	0.7
10.02 Birth asphyxia	1	6.7	3	3.9	0	0.0	0	0.0
03.03 Diabetes mellitus	0	0.0	0	0.0	0	0.0	2	1.4
06.02 Liver cirrhosis	0	0.0	0	0.0	0	0.0	2	1.4
08.01 Epilepsy	0	0.0	0	0.0	0	0.0	2	1.4
09.04 Obstetric haemorrhage	0	0.0	0	0.0	1	5	2	1.4
10.06 Congenital malformation	0	0.0	2	2.6	0	0.0	0	0.0
10.99 Other and unspecified neonatal death	0	0.0	2	2.6	0	0.0	0	0.0
12.01 Road traffic accident	0	0.0	0	0.0	0	0.0	2	1.4
03.01 Severe anaemia	0	0.0	0	0.0	0	0.0	0	0.0
05.01 Chronic obstructive pulmonary dis	0	0.0	0	0.0	1	5	1	0.7
07.01 Renal failure	0	0.0	0	0.0	0	0.0	1	0.7
10.04 Neonatal sepsis	2	13.3	1	1.3	0	0.0	0	0.0
12.04 Acid drowning and submersion	0	0.0	1	1.3	0	0.0	0	0.0
12.05 Acid expos to smoke fire & flame	0	0.0	0	0.0	0	0.0	1	0.7
99 Indeterminate	0	0.0	0	0.0	1	5	1	0.7
T50.9 Poisoning	0	0.0	0	0.0	1	5	0	0.0
B16.0 Hepatitis B	0	0.0	0	0.0	1	5	0	0.0
C22.0 Liver cell carcinoma	0	0.0	0	0.0	1	5	0	0.0
M22.9Systemic lupus erythematosus	0	0.0	0	0.0	1	5	0	0.0

## Discussion

Our results demonstrate that lay community health workers are able to conduct quality VA interviews to capture critical information that can be analyzed using standard methodologies to provide a more complete estimate of the burden and causes of mortality. This is due to the fact that findings conform to the patterns and causes of mortality which have been reported in other studies and trends similar to National level estimates [26]. A critical finding of this study was that more than half of all deaths (n = 123, 53.5%) occurred outside of the health facilities, and thus were not captured by the existing facility-based passive surveillance system. this is consistent with other reports from the region [26] and underpins the need for a functional community surveillance system which can help capture such deaths that go an accounted for. Additionally, there are important differences in the causes of death between those occurring in the community and those occurring in health facilities. The high number of out of the facility deaths could be explained by the limited access to health facilities due to the highly mountainous and extremely hard to reach by motorized transport nature of the sub county. Other factors like HIV AIDS-related stigma [27]. Lack of appropriate health care infrastructures like trauma centers [28] limited pediatric health services in lower health facilities [29] could be driving the lack of engagement with healthcare resources and contribute to the discrepancy between VA and facility mortality.

The substantially higher number of deaths which were identified using the VA approach compared with facility registers indicates a strong need to better understand health seeking behaviors in the general population, especially in the vulnerable age of under-five. While previous studies have employed community health workers in small pilot studies [30] or to augment surveillance using simplified VA tools [19, 20], our use of a validated WHO questionnaire over a large area is relatively novel and improves the generalizability of our findings. This success should not come as a surprise, as previous studies have shown that with training, community health workers can rapidly adopt the skills needed to perform VAs despite the lack of formal education[16]. Overall, we believe that this approach represents a promising and likely cost-effective means of improving the measurement of vital statistics in resource-limited settings with established community health workers infrastructure.

The potential public health implications of this study are important. First, our study shows that with appropriate training, community health workers can be mobilized to improve the collection of vital statistics and perform communicable disease surveillance. For example, with more frequent surveys, such an approach could track rapidly-spreading infectious disease epidemics, such as Ebola, Yellow Fever, or measles. Second, our results cast further doubt on the validity of existing passively collected data. A high proportion of deaths (n = 38, 52.1%) reported to have occurred in one of the three referral health facilities in the district could not be identified on review of the health facility records. This could be due to a number of factors, including incomplete or inaccurate recordkeeping inadequate health workers and weak health information systems and poor diagnostic infrastructure [31]. Additionally, the results show that a high number of deaths due to HIV/AIDS among children under five years occur in communities. This could be due to factors like lack of appropriate treatment for these children or late enrollment on care. Together, these results suggest that deaths in rural communities are greatly underreported, thereby exacerbating already stark urban–rural disparities. Lastly, our findings indicate a potential underestimation of the burden of non-communicable diseases (NCDs) and traumatic injuries, which were more commonly reported to have occurred outside of the formal health sector. If this pattern is consistent across rural sub-Saharan Africa, it may partly explain the long-standing under-appreciation of NCDs as a major cause of morbidity and mortality.

Our study has a number of strengths including the use of a validated VA tool and public-domain analytic software, relatively large sampling frame, and rigorous attempts at identifying deaths outside of the sub-county. Our study also has limitations. First, we did not directly compare the results of the VHT-administered VAs to results obtained by professional study staff. However, we are reassured by (1) the high quality of the directly observed encounters and [2] the general agreement in the leading causes of death identified with our survey and similar work conducted in other parts of Uganda. [32–35]. Second, we did not assess the feasibility of using community health workers to assign causes of death. Instead, we relied on study staff to enter the data into the InterVA tool. Thus, the issue of data entry and analysis will still need to be solved prior to implementation into routine surveillance programs. We assume these tasks will take place at the district level, but the ability of already overburdened health management teams to complete this work in a timely manner will need to be tested. We however believe that the recent release of the InterVA-5 model which is compatible with the WHO 2016 standard will make the task of analyzing verbal autopsy data much easier for the district statisticians. [31] Lastly, there is no reference standard for the true number of deaths occurring in the sub-county. Therefore, we cannot estimate the sensitivity of our approach for identifying deaths. Given that the survey recorded more than six times as many deaths as was reported to the District Health Office, there is little doubt that it represents a significant improvement over existing data collection systems.

## Conclusion

Community health workers can commendably conduct verbal autopsy interviews and generate valid data that can be used in health planning and policy formulation. This approach can be scaled to improve the measurement of vital statistics in order to facilitate appropriate public health interventions in rural areas of sub-Saharan Africa.

## Supporting information

S1 File (DTA)

S2 File (DTA)

S3 File (CSV)
